# High-grade sarcoma arising within a cellular dermatofibroma

**DOI:** 10.1016/j.jdcr.2024.12.003

**Published:** 2024-12-08

**Authors:** Nelson Ugwu, Nina Ran, Grant Fischer, Abigail Waldman

**Affiliations:** a Harvard Combined Dermatology Residency Training Program, Boston, Massachusetts; b Department of Dermatology, Brigham and Women's Hospital, Harvard Medical School, Boston, Massachusetts; c Department of Pathology, Brigham and Women's Hospital, Harvard Medical School, Boston, Massachusetts; d Veterans Affairs Integrated Service Network, Department of Dermatology, Jamaica Plain, Massachusetts

**Keywords:** dermatofibroma, dermatofibrosarcoma, malignant transformation, sarcoma

## Introduction

Dermatofibromas are a common entity encountered by dermatologists in everyday practice and are generally considered benign. Cellular dermatofibromas are a variant of dermatofibroma with a tendency to recur. Of note, malignant transformation of these lesions is rarely reported.^1^^,^^2^ Here, we describe a case of an elderly woman with a histologically confirmed cellular dermatofibroma that recurred with a high-grade sarcoma within.

## Case report

**Fig 1:**
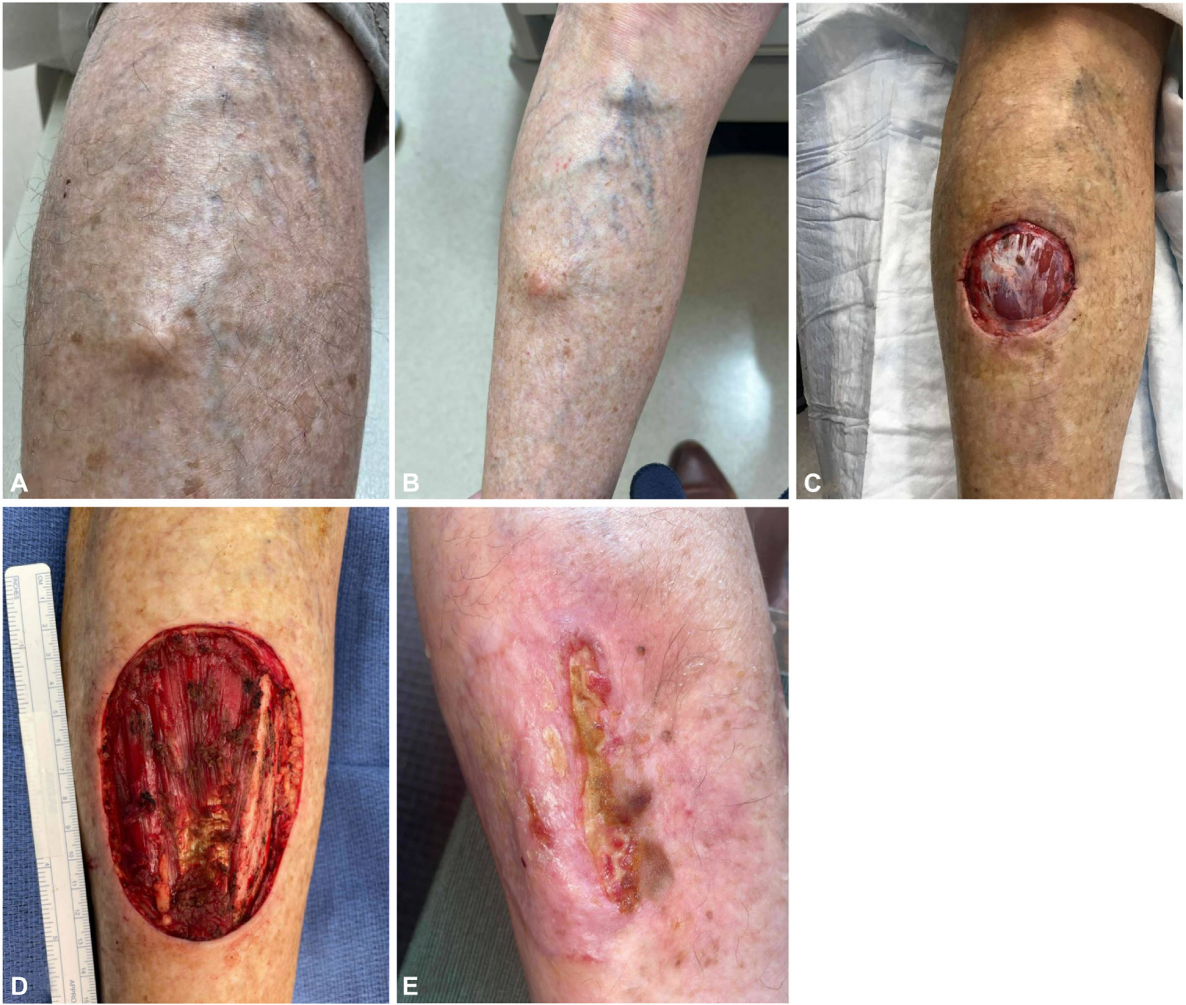
Clinical appearance of the lesion and defects following surgical intervention and subsequent reconstruction. **A,** On initial presentation, clinical examination revealed a 1.5 cm firm nodule on the right pretibial shin. **B,** The lesion recurred at the same site 18 months after excisional biopsy. **C,** A 4.9 cm surgical defect after the second stage of Mohs micrographic surgery, which was halted due to positive deep margins in fascia overlying tibialis anterior muscle. **D,** A 10 cm surgical defect following radical resection of the sarcoma with clear margins obtained. **E,** Surgical site 16 weeks following reconstruction, which consisted of a bipedicle flap with a split-thickness skin graft from the right thigh and the medial portion of the defect healing by second intention.

**Fig 2:**
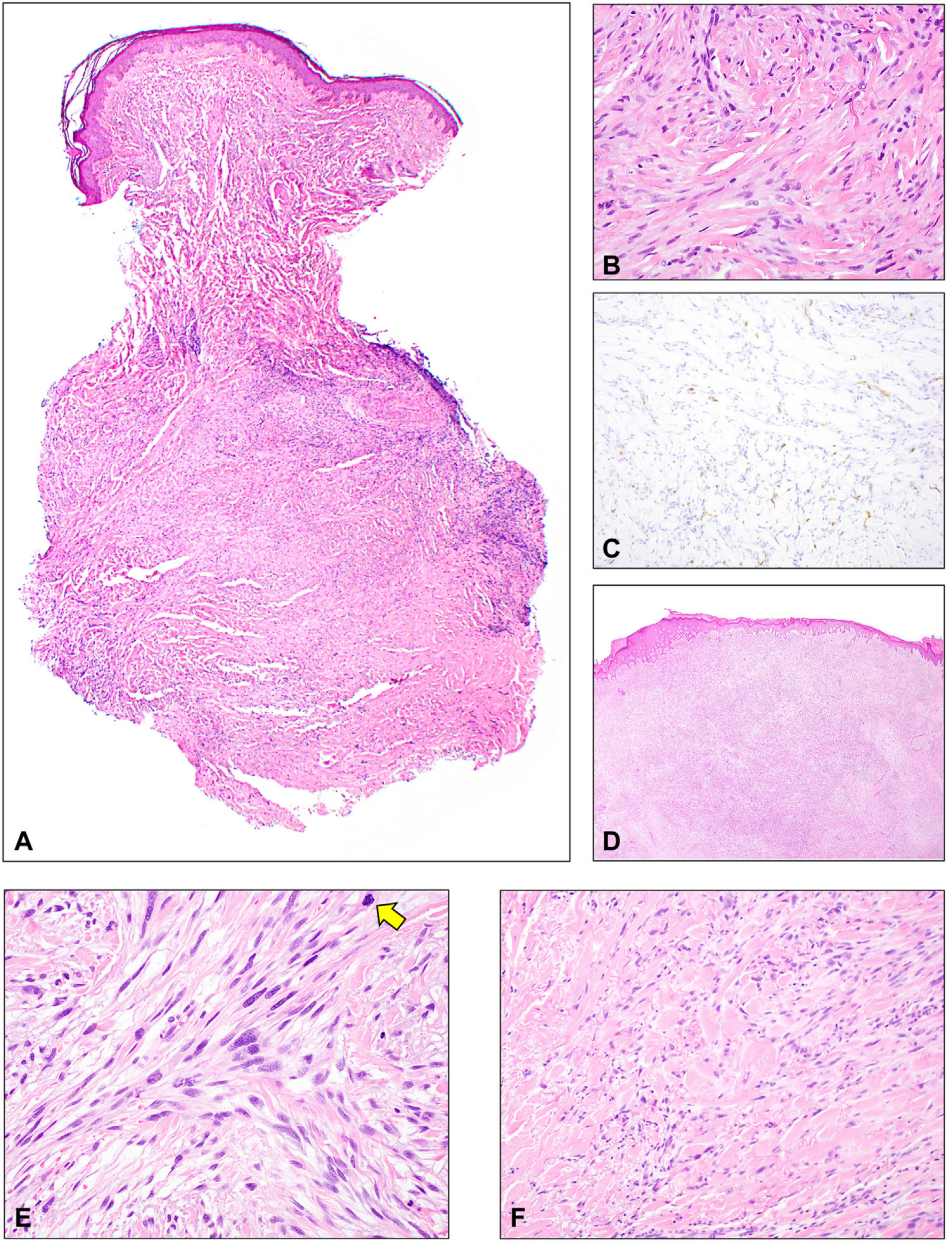
Histologic features from the cellular dermatofibroma and high-grade sarcoma. **A** and **B,** The cellular dermatofibroma consists of a deep dermal proliferation of cytologically bland spindled and histiocytoid cells arranged in a vaguely storiform pattern and with peripheral collagen trapping and subtle basal layer hyperpigmentation (H&E-stained slides, 20× magnification and 400× magnification, respectively). **C,** Tumor cells are without staining for CD34. Background vessels show variable staining (CD34 immunostain, 200× magnification). The tumor cells were also negative for desmin, SOX10, and HMB-45 immunostains (slides not shown). **D,** The high-grade sarcoma consists of a dense, infiltrative dermal and subcutaneous proliferation of cytologically atypical cells arranged in a variably fascicular and storiform pattern (H&E-stained slide, 20× magnification). **E,** The sarcoma cells are spindled in morphology and cytologically atypical with nuclear hyperchromasia and coarse chromatin. A mitotic figure is highlighted with the *yellow arrow* (H&E-stained slide, 400× magnification). **F,** Residual peripheral collagen trapping is present (H&E-stained slide, 200× magnification). The cells diffusely expressed α-SMA but were negative for desmin, SOX10, S100, AE1/AE3, CAM5.2, p63, and CD34 immunostains (slides not shown).

A 78-year-old woman presented to the dermatology clinic with a rapidly growing, asymptomatic subcutaneous nodule on the right anterior shin. Physical examination revealed a 1.5 cm firm, round, skin-colored subcutaneous nodule (Fig 1). Given the firm nature of the lesion, her dermatologist recommended imaging prior to removal to rule out deeper tissue involvement. A nonvascular ultrasound revealed a localized subcutaneous mass with overlying skin changes. The patient underwent an excisional biopsy 3 months after initial presentation, with histopathologic evaluation revealing a cellular dermatofibroma present at the specimen margins (Fig 2).

The patient noted regrowth of the lesion 18 months after her excisional biopsy. Clinical evaluation showed a 3 × 2.5 cm skin-colored, firm, immobile subcutaneous nodule at the biopsy site. Given the high recurrence rate associated with cellular dermatofibromas,^3^ as well as the rapid regrowth and large size of the lesion, the patient underwent surgical removal with Mohs micrographic surgery; however, the procedure was halted after the second stage due to positive deep margins in the fascia overlying the tibialis anterior muscle (Fig 1). The tumor was sent for histopathologic evaluation, revealing a high-grade sarcoma within the cellular dermatofibroma (Fig 2). Imaging was obtained to guide further management. Magnetic resonance imaging of the right tibia and fibula revealed a nodular 5 mm focus of enhancement along the base of the surgical defect. Following multidisciplinary discussions, she underwent neoadjuvant radiation (total dose of 50 Gy) followed by radical resection of the sarcoma by surgical oncology with clear margins 3 months after Mohs surgery (Fig 1). Reconstruction of the defect was then performed by the plastic and reconstructive surgery team, utilizing a bipedicle flap and split-thickness skin graft. A portion of the defect was left to heal by second intention (Fig 1). Eight months after radical resection of her sarcoma, the patient has remained free of recurrence.

Although it is remotely possible that an unsampled sarcomatous component existed in the original cellular dermatofibroma, this is unlikely given that the initial lesion was sampled through excisional biopsy. Cellular dermatofibromas are a variant of dermatofibroma with a higher tendency for subcutaneous extension and may recur in up to 10% of cases.^3^ However, malignant transformation of dermatofibromas, including cellular dermatofibromas, into sarcomas is exceedingly rare with only 3 cases reported in the literature.^1^^,^^2^ This case is unique in that it documents the development of a high-grade sarcoma arising within a histologically confirmed dermatofibroma. While dermatofibromas are considered benign, clinicians should maintain an index of suspicion for potential transformation and consider removal if these lesions recur, exhibit growth, or become symptomatic.

## Conflicts of interest

None disclosed.
